# Precision Public Health for Non-communicable Diseases: An Emerging Strategic Roadmap and Multinational Use Cases

**DOI:** 10.3389/fpubh.2022.854525

**Published:** 2022-04-08

**Authors:** Oliver J. Canfell, Kamila Davidson, Leanna Woods, Clair Sullivan, Noelle M. Cocoros, Michael Klompas, Bob Zambarano, Elizabeth Eakin, Robyn Littlewood, Andrew Burton-Jones

**Affiliations:** ^1^Centre for Health Services Research, Faculty of Medicine, The University of Queensland, Brisbane, QLD, Australia; ^2^UQ Business School, Faculty of Business, Economics and Law, The University of Queensland, Brisbane, QLD, Australia; ^3^Digital Health Cooperative Research Centre, Australian Government, Sydney, NSW, Australia; ^4^Health and Wellbeing Queensland, Queensland Government, The State of Queensland, Brisbane, QLD, Australia; ^5^Metro North Hospital and Health Service, Department of Health, Queensland Government, Herston, QLD, Australia; ^6^Department of Population Medicine at Harvard Medical School and the Harvard Pilgrim Health Care Institute, Boston, MA, United States; ^7^Department of Medicine, Brigham and Women's Hospital, Boston, MA, United States; ^8^Commonwealth Informatics Inc., Waltham, MA, United States; ^9^School of Public Health, Faculty of Medicine, The University of Queensland, Brisbane, QLD, Australia

**Keywords:** electronic health records, medical informatics, non-communicable diseases, preventive medicine, public health, public health informatics, precision public health, electronic medical records

## Abstract

Non-communicable diseases (NCDs) remain the largest global public health threat. The emerging field of precision public health (PPH) offers a transformative opportunity to capitalize on digital health data to create an agile, responsive and data-driven public health system to actively prevent NCDs. Using learnings from digital health, our aim is to propose a vision toward PPH for NCDs across three horizons of digital health transformation: Horizon 1—digital public health workflows; Horizon 2—population health data and analytics; Horizon 3—precision public health. This perspective provides a high-level strategic roadmap for public health practitioners and policymakers, health system stakeholders and researchers to achieving PPH for NCDs. Two multinational use cases are presented to contextualize our roadmap in pragmatic action: *ESP* and *RiskScape* (USA), a mature PPH platform for multiple NCDs, and *PopHQ* (Australia), a proof-of-concept population health informatics tool to monitor and prevent obesity. Our intent is to provide a strategic foundation to guide new health policy, investment and research in the rapidly emerging but nascent area of PPH to reduce the public health burden of NCDs.

## Introduction

### Background

Non-communicable diseases (NCDs) are a significant global health challenge (1). People of all ages and countries are affected by NCDs; together, they contribute to 71% of all deaths globally (2). We are now living amidst a global syndemic—a synergy of three parallel, co-occurring epidemics—of NCDs, social inequity and coronavirus disease (COVID-19) which is highlighting the limitations of traditional public health models to address burgeoning risk factors and disease (3–6). For example, malnutrition (including obesity and undernutrition) is now the leading risk factor and cause of poor health globally (7), including the four major NCDs (8): cardiovascular disease (CVD), cancers, chronic respiratory conditions and diabetes, as well as mental health (9).

In the digital era, jurisdictions have the opportunity to reimagine public health approaches to address NCDs. Precision public health (PPH) seeks to create an agile, responsive and data-driven public health system that strengthens current evidence-based approaches. PPH aims to improve population health by integrating data and digital technology to guide precision decisions, interventions and policy (10, 11). From a digital perspective, the foundation of creating a PPH system is harvesting “organic” data that is collected automatically, routinely and in real-time *via* digital health infrastructure (e.g., electronic medical/health records, personal health records, genomics, billing/claims, mobile health, digital wearables, social media) (11, 12). From a public health perspective, organic data is repurposed data (from clinical or social systems, as examples) to improve public health and is *not* collected through a specific research design (13).

In contrast, current public health practice uses “designed” data that is created using specialized methods by the user, often as retrospective point-prevalence disease snapshots, administrative databases and disease registries (12). Designed data is bespoke—it has been designed for a specific purpose and is often single-use, or repeated-use for the same purpose. Harvesting organic data has advantages of volume, contemporaneity and granularity and can thus improve decisions based upon designed data (11). The U.S. National Health and Nutrition Evaluation Survey (NHANES), for example, only includes a few thousand people per year whereas emerging surveillance systems leveraging electronic health record data typically include millions of people.

Using organic data for PPH has matured for communicable diseases (e.g., cholera, influenza, and Zika virus) and COVID-19 has accelerated this transformation (14, 15). Fast action against communicable disease requires access to fast (organic) data from consumer-centered sources—transmission happens in real-time and public health responses must be rapid to manage the acute threat. One example is the Digital Coronavirus Application (DCOVA), developed to capture real-time information from digitally-enabled public health systems about people required to quarantine in Queensland, Australia to enable precision tracking and monitoring (16). Digital health transformation of public health to manage communicable diseases offers a starting blueprint but unique considerations must be made for NCDs. The data requirements are diverse as NCDs are driven by a complex system of social, environmental, behavioral, biomedical and commercial determinants and NCD prevalence can change slowly over the lifecourse. The social, environmental, and behavioral determinants primarily exist in communities where, historically and compared to the acute healthcare sector, there has been little investment in digital health and analytics and so surveillance and targeting interventions has been difficult (11, 17–19).

The maturing digital era of electronic medical/health records (EMRs/EHRs), personal health records (PHRs), mobile health (mHealth), wearables, Internet of Things (IoT), social media and genomics can offer a richer understanding of community-based NCDs that leads to improved preventive decision-making. Digitally-enabled public health interventions are emerging in interventional literature (20). For PPH of NCDs, an example of using organic data is just-in-time adaptive interventions (JITAIs) where prescriptive physical activity recommendations are “pushed” to individuals based on real-time monitoring of sedentary behavior *via* wearables (20).

### Strategic Momentum for Precision Public Health

The rapid and disruptive digital transformation of healthcare is the strongest enabler to PPH. In 2014, this was recognized by coining the “learning public health system,” where data and digital technology is used to improve prevention, public health practice and outcomes (an early conceptualization of PPH and sister concept to the “learning health system”) (21, 22). The PPH term was then introduced in academic literature in 2015 to propagate a modernization of information systems to enable better surveillance and targeted interventions (23). From 2015, research accelerated in the use of EHRs for population health and disease surveillance (24–26), and in developing a national (USA) vision for population health informatics (27). Concurrently, the Topol Review (2018) proposed public health informatics as a strategic area to bridge digital medicine with public health and disease prevention (28).

In global response, the European Public Health Association (EUPHA) published a vision for “digital public health” in Europe to support the transition from cure to prevention (29), and the Pan American Health Organization (PAHO) described their eight principles for digital transformation of healthcare (30, 31). In Australia, an argument for digital health transformation toward PPH was proposed in 2021 across three conceptual “horizons” (11):

- Horizon (1) Building digital health prevention foundations (*digital public health workflows*)
- Horizon (2) Transforming preventive decisions using data and analytics (*population health data and analytics*)
- Horizon (3) New models of preventive care (*precision public health*).

### Vision and Aims

This article aims to leverage global strategic momentum for precision public health and operationalize the three horizons for digital health transformation toward a unified vision—“*Precision public health to prevent non-communicable diseases*.” To achieve our aim, we:

1. Propose a strategic roadmap that is internationally relevant to public health and health system professionals, policymakers, consumers and communities, and researchers.
2. Describe two use cases—one mature (USA) and one emerging (Australia) to ground our roadmap in action and offer real-world utility for other jurisdictions.
3. Discuss the current state and future state of PPH for NCDs, and offer lessons learned from our use cases to guide relevant stakeholders.

## Methods

To develop the roadmap, we reviewed and integrated salient literature across two areas: (1) digital transformation of public health (emerging) and (2) digital transformation of the healthcare sector (advanced). The three horizons were applied as a conceptual framework to organize key literature themes into “objectives” and thus present a structured roadmap. The roadmap was finalized after including subject matter expert contributions and reaching consensus. The vision, methods and characteristics of multinational (USA and Australia) use cases are then presented as practical applications of the strategic roadmap.

## Results

### Strategic Roadmap—Precision Public Health for Non-communicable Diseases

**Figure 1:**
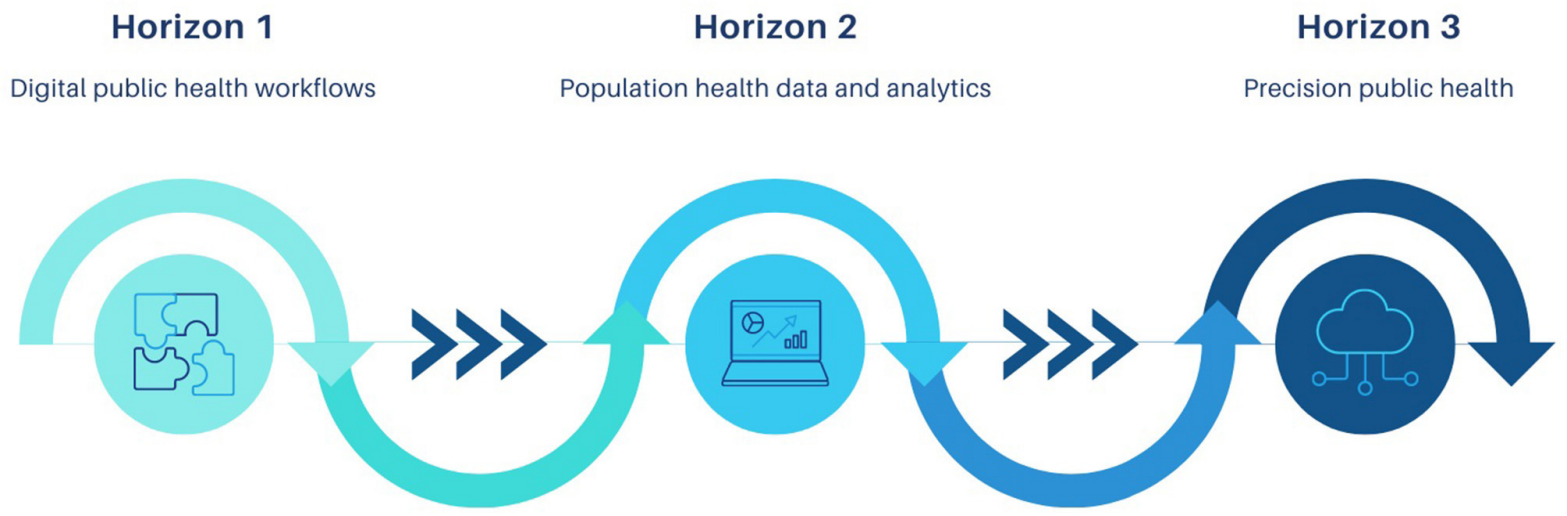
Three horizons roadmap for precision public health of noncommunicable diseases.

This roadmap (see Figure 1) presents emerging objectives for strategic action, investment and research across the three horizons. Stakeholders can self-assess their progress—dependent upon local context—against the three horizons to modulate their strategic direction appropriately.

### Horizon 1—Digital Public Health Workflows

Horizon 1 focuses on building the necessary digital infrastructure, workflows and partnerships as the foundation for creating PPH models of public healthcare.

#### Objectives

##### Map Useable Data Assets

Public health practitioners and policymakers must identify and evaluate useable “designed” and “organic” data assets for PPH of NCDs in their local, state or national jurisdictions (32). Evaluation must comprise the (a) availability and (b) quality of the data asset (32). Mapping designed data assets provides an assessment of current state and mapping organic data assets provides a vision for the future state (i.e., PPH).

##### Build Prevention Partnerships Across Sectors

Generate high-trust partnerships between government sectors that influence the determinants of NCDs, such as healthcare, education, agriculture, sports, transport, urban infrastructure and academia. Identify and appoint key organizational champions to support an overarching PPH for NCDs vision. Population health analytics tools were found to be more successful if enabled by partnerships across academia, public health agencies, and healthcare as a minimum (32).

##### Digitize Public Health With Organic Data Assets

Harvest organic health data (EMRs/EHRs, PHRs, genomics, billing/claims, mHealth, IoT, wearables, and social media) within public health organizations as the data foundation for PPH (32). For example, in Australia, leverage a strong healthcare sector partnership to prioritize local and state EMRs/EHRs as the core data asset to ensure adequate population reach, data granularity, real-time data capability and coverage of all determinants of NCDs (32). In the USA, the Public Health Data Warehouse in Massachusetts aggregates multiple organic sources to track the opioid epidemic and maternal and child health inequities (33).

##### Develop Digital Public Health Literacy

Public health practitioners need specialized training in data science to support predict-prevent models of public healthcare (10, 11, 28). Learnings from the acute healthcare sector emphasize that the key requirements for successful digital transformation must include digital governance, workforce training and upskilling, cultural readiness and transparency (34).

### Horizon 2—Population Health Data and Analytics

In Horizon 2, analytics are applied to data from Horizon 1 to transform and better target preventive decisions. The primary goal is to create data-driven decision-making that improves upon standard public health models of care.

#### Objectives

##### Develop Digital Infrastructure to Support Informatics

Aggregate consumer-centered data from designed (e.g., large health surveys) and organic sources (e.g., EMRs and billing/claims) and create a single source of truth. Interoperability standards remain a challenge and aggregating various data with a common unique health identifier is a potential solution (25). Draw this data into a business intelligence layer to create visualizations. Population data visualizations positively influence public health practitioner experience and self-efficacy (35). Data visualizations mean practitioners can quickly evaluate population health data and easily interpret meaning (i.e., what are the socioeconomic risks in *x* suburb where obesity prevalence is highest?).

##### Descriptive, Predictive, and Prescriptive Analytics

Start simple and overlay diverse descriptive analytics to understand the target population. Geospatial, comparative and temporal functions can be applied first to characterize NCD prevalence and changes over time to assist monitoring and evaluation. Predictive analytics can then be applied on a community and population-level to estimate long-term disease risk to target interventions and resourcing. Prescriptive analytics (e.g., a decision rule) can provide decision-support to public health practitioners and policymakers based on key parameters, for example, a school-based healthy lifestyle intervention is needed in *x* community with high childhood obesity prevalence, lower educational attainment and higher cost of healthy living.

##### Digital Governance and Data Privacy

Appropriate data and digital governance become critical as the public health workforce diversifies and workflows begin to transform. A bottom-up, tiered and transdisciplinary governance approach is required to effectively plan and manage for digital transformation (36). Population health informatics must operate on non-identifiable, aggregate data to protect consumer privacy. Multi-level stratifications (e.g., by hospital facility AND suburb AND diabetes status) in data visualizations must be monitored to ensure data cannot re-identify individuals.

### Horizon 3—Precision Public Health

Horizon 3 creates new, digital-first models of PPH for NCDs that adopts a learning “predict-prevent” system based on a granular understanding of NCD risk. As new PPH models of care succeed or fail, the data that informed their design changes as the health of the population changes. To truly achieve PPH the digital systems created in Horizon 1 and Horizon 2 must inherently *learn* to modify risk and interventions according to changes in population health.

#### Objectives

##### Build an Evidence-Base for New Digital Models of Prevention

Testing the effectiveness of PPH interventions is challenging as traditional study designs (e.g., randomized controlled trials) are not fit-for-purpose for a “predict-prevent” and highly responsive public health system that must measure responses in communities and populations, rather than individuals (10). PPH interventions, such as JITAIs that push tailored nutrition advice based on dietary intake recorded on a mobile app, must be evaluated using alternative study designs (e.g., n-of-1 and microrandomised trials) (10, 17) as they are yet to be sufficiently evidenced to warrant integration in core public health practice, especially for vulnerable groups. The Quadruple Aim of Healthcare (37) (improving patient/consumer experience, improving clinician/practitioner experience, better outcomes and lower cost) can be adapted to public health to provide a clear framework for building the evidence-base for PPH and justifying investment.

##### Leverage Digital to “Count” Preventive Impact

It is difficult to incentivize and reward PPH models of care when population health is currently not measured in a contemporaneous, granular way. Using organic data will help to create methods of “counting” prevention, as consumer-centered data is available in real-time. Measuring preventive impact must shift from a negative focus on burden (e.g., disease prevalence, unhealthy behaviors) to positive measures of health and wellness. One new public health deliverable may be improvements in healthier on-the-spot purchasing behavior based on a digital “push” to a mobile app. Prevention must be rewarded but this cannot be realized without measures of success.

##### Invest in Change Management of the Public Health Sector

Implementing Horizons 1–3 will create a disruptive shift in public health organizational and practitioner culture. For example, a key function of a public health practitioner may change from long-term planning (based on old designed data) to reactive, responsive planning (based on organic data). Public health informaticians and managers will need change leadership capabilities to successfully advance institutional structures toward PPH, and manage tensions whilst implementing models of PPH care.

### Multinational Use Cases

**Figure 2:**
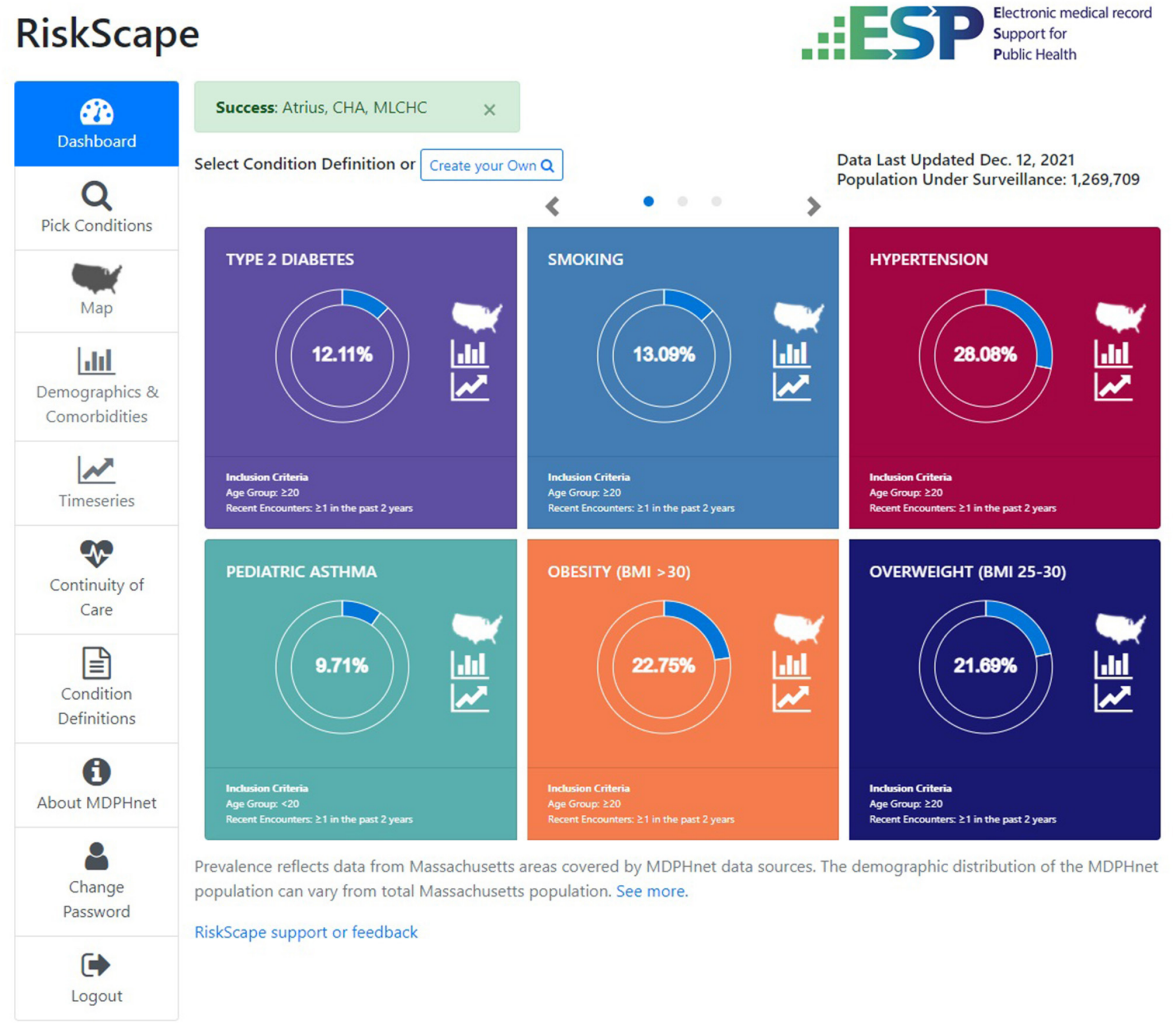
RiskScape data visualisation platform (USA).

**Figure 3:**
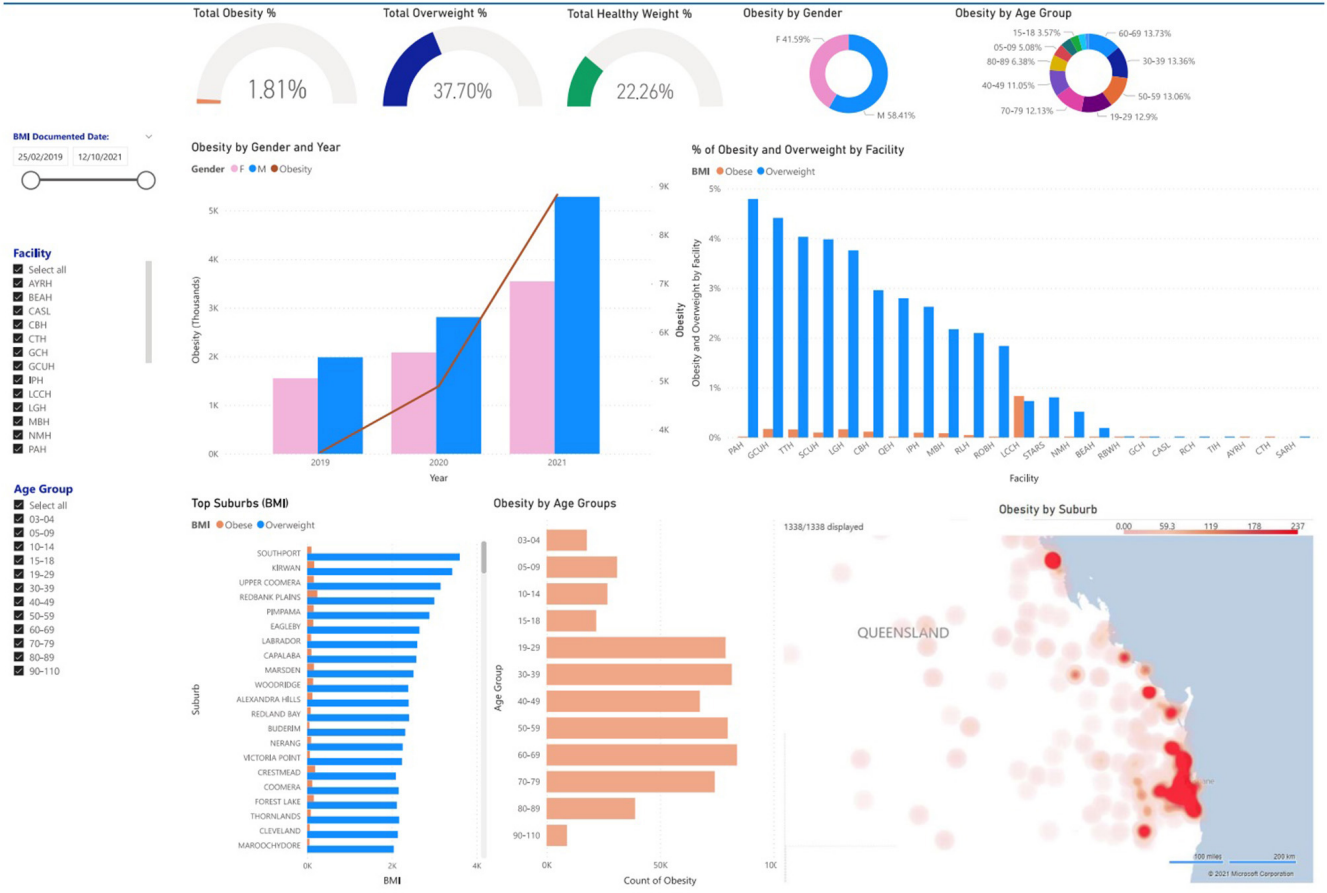
Population Health Queensland (PopHQ) proof-of-concept (Australia).

Table 1 presents the key elements of *ESP* and *RiskScape* and *PopHQ* across domains of digital health transformation: (a) People, (b) Process, (c) Information, and (d) Technology (38). Figure 2 presents *RiskScape* and Figure 3 presents *PopHQ*.

**Table 1:** Key elements of two multinational use cases for precision public health of NCDs—*RiskScape* (USA) and *PopHQ* (Australia).

**Domain**	**Element**	**RiskScape**	**PopHQ**
People	Setting	Massachusetts, USA	Queensland, Australia
	Population (sample)	~1.2 million	~1 million
	Population (cohorts)	Ages 0 through >80*	0–1, 1–2, 2–5, 5–12, 12–17, and 18–65 years
	Target disease/s	NCDs (e.g., diabetes, body mass index categories, asthma, treated depression, cardiovascular risk score categories, hypertension), CDs (e.g., influenza-like illness, pertussis syndrome), smoking status, immunizations, laboratory testing (e.g., triglycerides, cholesterol), use of opioids and benzodiazepines	Obesity
	Developers	State public health, academic epidemiologists, clinical partners, informaticians	Medical, allied health, informaticians, data analyst, business analyst, software engineer, researcher
	Users	State public health and participating clinical partners	Public health practitioners and policymakers, health system planners and managers, researchers
Process	Data extraction	Any of several EMR-specific SQL ETL processes, or ETL from other standard clinical data structures (e.g., OMOP, PCORI), or HL7 CCDA format, or FHIR bulk data extract (in development)	Data extraction wizard used to process EMR metadata into a business intelligence layer
	Governance	A single governance rules document was developed and agreed to by all network members. Network members meet on a monthly basis.	Data sharing agreement between healthcare sector (EMR) and public health sector (users)
Information	Source	EMR	EMR
	Data elements	Annual encounters, clinical practice, age, race, Hispanic ethnicity, height, weight, blood pressure, relevant diagnoses, prescriptions, immunizations, and laboratory results	Last encounter, facility, age, country of birth, suburb, height, weight, body mass index (BMI)
	Refresh frequency	Monthly	Quarterly
	Privacy	Non-identifiable, aggregated metadata	Non-identifiable, aggregated metadata
Technology	Visualization	Backend: Linux, PostgreSQL App layer: Python, Django, Nginx, Gunicorn, Redis, Supervisor, Frontend: Bootstrap4, d3.js, c3.js, leaflet.js, carto-db, font-awesome, custom jquery	Visual engine—PowerBI
	Analytics	Descriptive (geospatial, comparative, temporal)	Descriptive (geospatial, comparative, temporal)

*EMR, electronic medical record; BMI, body mass index*.

* *0–30 in 5 year groups, then 30–80 in 10 year groups, then over 80*.

#### ESP and RiskScape (USA)

The first use case (USA) is well-developed and describes a mature population health informatics tool, *ESP*, and its related data visualization platform, *RiskScape* (39).

##### Vision

**E**lectronic medical record **S**upport for **P**ublic Health ESP is an open-source software platform that organizes and maps organic EMR data, analyzes the data for conditions of public health interest (including communicable and non-communicable diseases), and can automatically transmit individual case reports or aggregate summaries to health departments (esphealth.org) (40–42). *ESP* has been implemented in a variety of jurisdictions in the US for case-based reporting of communicable diseases or adverse vaccine events. *ESP* is also used for chronic disease surveillance in multiple US jurisdictions and serves the foundation for the Multistate EHR Network for Disease Surveillance (MENDS) system, a pilot project sponsored by the US Centers for Disease Control and Prevention and organized by the National Association of Chronic Disease Directors to create a demonstration national electronic surveillance system for chronic diseases (43).

*RiskScape* is *ESP*'s interactive, web-based data visualization platform. It provides timely, high-level summaries of specific conditions of interest to public health officials including NCDs like asthma, hypertension, smoking, obesity, and diabetes (39). *RiskScape* utilizes data from clinical partners that participate in *ESP*, but is built on a centralized, individual-level, deidentified dataset that is updated monthly.

##### Methods

The current capabilities in *RiskScape* include heat maps by zip code; stratifications by demographics and comorbidities; time series analyses with trend statistics; diabetes, HIV and HCV continuums of care; and care cascades among those at high risk of HIV (e.g., the proportion that receive pre-exposure prophylaxis) and cardiovascular disease.

*RiskScape* has been fully implemented to support the Massachusetts Department of Public Health and participating clinical practices by providing timely, easy to access insight into the prevalence of selected conditions as well as various aspects of clinical care among patients of interest, and patterns in testing (e.g., cholesterol, hemoglobin A1C). It also enables identification of potential health disparities. *RiskScape* unloads analytic burden from health department and clinical staff and centralizes instead in an efficient, optimized electronic environment. In addition, clinical practices can gain understanding into disease patterns and management practices beyond their own patients and into their catchment area as *RiskScape* integrates data from multiple practices. Currently, the instance of *RiskScape* in Massachusetts covers ~20% of the state population, >1.2 million individuals.

As we look ahead to what is possible with *RiskScape* to further support PPH, possibilities include implementing more risk prediction tools for undiagnosed conditions or lapses in care, cascades of care that allow drill down to identify individual patients with care opportunities, integration of additional social determinants of health, and more sophisticated risk adjustment tools to adjust for differences between observed vs. total populations for a given jurisdiction.

#### PopHQ (Australia)

##### Vision

The second use case (Australia) presents a proof-of-concept, still in development population health informatics tool, *PopHQ*, to specifically address obesity prevention and public health in a large state, Queensland (population >5 million).

The vision for *PopHQ* is to harvest anonymized clinical EMR data (Horizon 1) and apply descriptive analytics (Horizon 2) to create a useable tool to guide precision decision, investment, interventions and resourcing in public health agencies—such as the first dedicated prevention health agency in Queensland (44)—and the state's health system. Anonymized EMR data will be extracted from Queensland's integrated-electronic medical record (ieMR) that services over 70% of its public health system. It is the largest single EMR instance in Australia. The proof-of-concept *PopHQ* is presented in Figure 3 and is currently presented as a ‘mock-up' without using patient data. The final total sample size will be ~5 million.

##### Methods

Stakeholders across the healthcare sector, public health sector and academia attended a design workshop to provide feedback on the purpose, interface and overall design of *PopHQ*. Stakeholders were asked (1) what do you like? (2) what don't you like? (3) what could be improved? A user story format was then used to identify how a stakeholder could use *PopHQ*—“as a <role> I want to <capability> so that <receive benefit>” e.g., as a public health practitioner, I want to see heat maps of overweight and obesity in Queensland geographic areas so I can target interventions.

Stakeholders identified various uses of *PopHQ* to create their user stories, including the ability to: compare obesity across age groups to target interventions across the lifecourse (public health practitioner); see obesity counts by facility to direct appropriate resourcing (systems planner); see counts and percentages of obesity in Queensland to justify the problem (researcher); stratify by suburb and facility to compare obesity across Queensland (generic user).

Despite the capability of *PopHQ* to update in real-time, the utility of this is low as obesity emerges and changes slowly over time. The dashboard will be updated quarterly to better represent trends in obesity and help granularize financial decisions.

## Discussion

This article presents a strategic roadmap to implementing three horizons for digital health transformation toward PPH for NCDs illustrated by multinational use cases. Global public health and health system professionals, policymakers, consumers and communities and researchers can translate this roadmap to their local jurisdictions to help guide organizational strategy, investment, intervention design and research.

### Current State of Precision Public Health for Non-communicable Diseases

The current global state is an amalgamation of progress across Horizon 1 and 2, primarily to create precision surveillance platforms for NCDs, rather than PPH models of care (Horizon 3). The rapid uptake of EMRs/EHRs into healthcare has already advanced population health surveillance for NCDs including asthma, smoking and diabetes (24). Contemporary population health informatics tools for NCDs are built upon organic (billing/claims, retail transactions, social media) and designed (surveys, administrative datasets) data together (45, 46). The primary purpose of these tools is precision surveillance of NCDs; most apply descriptive analytics to characterize local or state disease burden. These are promising steps; however, there are limited examples of mapping useable data assets for PPH of NCDs, building digital public health workforce literacy, and how to establish multi-sectoral prevention partnerships with the shared goal of PPH.

### Toward Precision Public Health for Non-communicable Diseases

The core gap between current digitally-enabled public health practice and PPH is advancing from precision surveillance (Horizon 2—using data to monitor disease) to precision action (Horizon 3—using data to build new digital-first public health models of care). Precision action is enabled by maturing (a) data (b) analytics and (c) an evidence-base for PPH interventions. Data must leverage diverse organic sources, analytics must advance to predictive and prescriptive capabilities and there must be evidence-based interventions to implement when indicated by supportive analytics.

Traditional public health principles must remain core to a PPH future (18). First, consumer and community voices will be central to managing potential implications of PPH on privacy, data misuse and security. Using experiential and participatory, bottom-up co-design approaches for PPH will help to build public trust for PPH interventions and normalize the digitization of public health (47). Second, precision must be used to advance equity, not inequity. Precision has been discussed as useful to granularize *social context* by identifying (a) precise social positions of populations (b) enhance measurement of social position (18), and to uncover pockets of inequalities in geographically small areas by analyzing population health at a high spatial resolution not at a state or national level (48). Third, intersectoral collaboration that is refocused toward PPH is required and will eventuate if driven by political will to improve data-driven approaches to public health, especially for our most vulnerable communities.

### Lessons Learned

*ESP* was initially developed in Massachusetts in 2005 with funding by the US Centers for Disease Control and Prevention for automated, electronic communicable disease reporting. Strong relationships between the state health department, the academic department that developed the system and the clinical practices were imperative to the system's initiation. Over time *ESP*-based case reporting in Massachusetts has expanded considerably, includes aggregate-level NCD surveillance, and has been adopted by other jurisdictions. It has been used to monitor community- and clinic-level interventions as well as provide general information on NCD prevalence. We developed *RiskScape* to make a subset of the EMR data within *ESP* quickly and easily accessible to users from a variety of perspectives—geography, time and patient subgroups. The inclusion of “care continuums” into *RiskScape* (e.g., to understand the proportion of patients with the presence of or risk for cardiovascular disease with various labs of interest and unmeasured hypertension) bring us closer to realizing the potential for PPH. In Massachusetts, this system was built gradually over time with a continuous commitment to strong governance and trust among all stakeholders. From this model of governance and stakeholder participating, *RiskScape* has been implemented in multiple states across the US for a federally funded pilot project (43). We are also considering a new model of *RiskScape* that would be built on data with patient identifiers, enabling participating clinical practices to drill down on high-risk groups to those specific patients needing outreach (e.g., patients at high risk for HIV who have not been tested or who have not been prescribed pre-exposure prophylaxis).

The *PopHQ* use case highlights the strategic value of uniting multiple sectors (healthcare, public health and academia) toward the shared vision of PPH for NCDs. The development of *PopHQ* was declared an operational activity for the healthcare sector as opposed to a research priority for the public health or academic sectors. A simple use case was proposed (i.e., mapping routinely collected clinical BMI across the state) to manage any disruption and to create a “minimum viable product” (MVP). Once successfully implemented, this simple MVP could then be upscaled to greater complexity (e.g., nutrition-based data elements, integrating with primary health care datasets). Ultimately, a key lesson in messaging to stakeholders was communicating the public health opportunity of sharing big EMR data to achieve PPH and improve disease prevention is too promising to ignore.

## Conclusion

There is significant potential for PPH to advance population health equity, reduce NCD risk and prolong healthy life (14, 15). Public health organizations and policymakers must capitalize on the global digital transformation of health to challenge the current models of addressing NCDs. We offer a clear roadmap across three Horizons to assist strategy, investment, intervention planning and research for PPH of NCDs and ultimately achieve equitable health and wellbeing for our populations.

## Data Availability Statement

Due to data privacy restrictions, data is not available. Requests to access these datasets should be directed to o.canfell@uq.edu.au.

## Author Contributions

OC, AB-J, and CS conceptualized the manuscript. OC and KD drafted the manuscript. NC, MK, and BZ contributed the USA use case, lessons learned, and critically reviewed the draft manuscript. LW, CS, EE, and RL critically reviewed iterations of the draft manuscript. All authors reviewed and approved the final manuscript.

## Funding

OC was funded by the Digital Health Cooperative Research Center, Australian Government(DHCRC-0083).

## Conflict of Interest

BZ was employed by Commonwealth Informatics Inc. The remaining authors declare that the research was conducted in the absence of any commercial or financial relationships that could be construed as a potential conflict of interest.

## Publisher's Note

All claims expressed in this article are solely those of the authors and do not necessarily represent those of their affiliated organizations, or those of the publisher, the editors and the reviewers. Any product that may be evaluated in this article, or claim that may be made by its manufacturer, is not guaranteed or endorsed by the publisher.
